# Strain variation in response to lung ischemia: role of MMP-12

**DOI:** 10.1186/1465-9921-13-93

**Published:** 2012-10-12

**Authors:** Clarke G Tankersley, Aigul Moldobaeva, Elizabeth M Wagner

**Affiliations:** 1 Department of Environmental Health Sciences, Bloomberg School of Public Health, Johns Hopkins University, Baltimore, USA; 2 Department of Medicine, School of Medicine, Johns Hopkins University, Baltimore, USA; 3 Johns Hopkins Asthma and Allergy Center, Division of Pulmonary and Critical Care Medicine, 5501 Hopkins Bayview Circle, Baltimore, MD, 21224, USA

**Keywords:** Angiogenesis, Chemokines, DBA/2J, C57BL/6J, Matrix metalloproteinases, Neovascularization

## Abstract

**Background:**

Systemic neovascularization of the lung during chronic ischemia has been observed in all mammals studied. However, the proteins that orchestrate the complex interaction of new vessel growth and tunneling through lung tissue matrix have not been described. Although previous work has demonstrated the CXC chemokines are essential growth factors in the process of angiogenesis in mice and rats, key matrix proteins have not been identified.

**Methods:**

Since the degradation of chemokines has been shown to be dependent on metalloproteinases (MMP), we first surveyed gene expression patterns (real time RT-PCR) of several lung matrix proteins in DBA/J (D2) mice and C57Bl/6 (B6) mice, strains known to have divergent parenchymal responses in other lung disease models. We studied changes in the time course of MMP-12 activity in D2 and B6 mice. Functional angiogenesis was determined 14 days after the onset of complete left lung ischemia induced by left pulmonary artery ligation (LPAL), using fluorescent microspheres.

**Results:**

Our results confirmed higher levels of MMP-12 gene expression in D2 mice relative to B6, which corresponded to a phenotype of minimal systemic angiogenesis in D2 mice and more robust angiogenesis in B6 mice (p < 0.01). MMP-12 activity decreased over the course of 14 days in B6 mice whereas it increased in D2 mice (p < 0.05). MMP-12 was associated largely with cells expressing the macrophage marker F4/80. Genetic deficiency of MMP-12 resulted in significantly enhanced neovascularization (p < 0.01 from B6).

**Conclusion:**

Taken together, our results suggest macrophage-derived MMP-12 contributes to angiostasis in the ischemic lung.

## Background

Systemic angiogenesis occurs in mice during chronic pulmonary ischemia after complete left pulmonary artery obstruction [1]. The global gene expression profile and subsequent protein validation demonstrated that the ELR + CXC chemokines are upregulated early after the onset of ischemia, and are essential to the process of systemic neovascularization of the lung [2,3]. These observations provide further support to the accumulating body of evidence demonstrating that the pro-angiogenic chemokines, MIP-2α, KC, and LIX (macrophage inflammatory protein-2 alpha, mCXCL2; keratinocyte-derived chemokine, mCXCL1; and lipopolysacharide-induced chemokine, mCXCL3, respectively) in the mouse contribute to the process of new systemic vessel growth and invasion into pulmonary parenchyma [4-6]. However, proteases responsible for degradation of these key growth factors are unknown with respect to the overall angiogenic response.

Interestingly, Dean and colleagues [7] have shown that several CXC chemokines are degraded by matrix metalloproteinases (MMP). Specifically, macrophage-derived matrix MMP-12 (macrophage elastase) was shown to cause proteolysis at the terminal E-LR (Glu-Leu) motif and inactivate both MIP-2α and KC, but not LIX [7]. MMP-9 (gelatinase B) was shown to increase the chemotactic activity of IL-8 (CXCL8) 10-fold in human tissue by amino-terminal processing [8]. The metabolic processing of growth factors by MMP is not unique to the CXC chemokines as Ebrahem and colleagues showed that MMP-9 was essential for release of sequestered VEGF, but was also involved in the proteolysis of this ubiquitous growth factor [9]. Additionally, MMP-12 has been shown to be critical to the process of plasminogen degradation into the anti-angiogenic factor angiostatin [10]. These observations highlight the importance of MMP in regulating the activity of key proteins that impact neovascularization. However, the role of MMP in modulating angiogenesis has long focused on their importance in degrading extracellular matrix in advance of new vessel growth [11,12]. In this capacity, the MMP family of zinc binding, Ca^2+^-dependent neutral endopeptidases can act individually or together to degrade extracellular matrix. Of these proteins, MMP-2 (gelatinase A) and MMP-9 have been shown to work in tandem and appear to be essential during the early stages of angiogenesis in several models of hypoxic, ischemia-induced angiogenesis to promote tissue remodeling as well as growth factor activation [11,13]. Plasma proteins and the tissue inhibitors of metalloproteinases (Timp) are key endogenous regulators of MMP. A variety of other matrix proteins and protein fragments have been shown to induce both inflammation and angiogenesis in several models of ischemia-induced neovascularization [14,15].

Given the inherent complexity of matrix changes during tissue ischemia, in vivo models may appear impenetrable. However, examination of mouse strain-dependent differences in gene expression patterns has yielded important information concerning complex changes in the lung during aging [16,17], injury and repair [18] and lung provocation [19-21]. Previous reports describe a difference in matrix gene expression patterns between DBA/2J (D2) and C57BL/6J (B6) mice in aging lung [16,17]. Consequently, in the current study, we used a similar strategy and surveyed changes in a panel of matrix-relevant genes in different mouse strains during ischemia. We subsequently focused on MMP-12 activity early and late in the process of neovascularization to determine potential mechanisms of response. Our results demonstrate that there are substantial differences in the rate of lung angiogenesis between B6 and D2 strains and that MMP-12 is overall angiostatic during lung ischemia.

## Methods

### Animals

Male DBA/2J (D2) and C57BL/6J (B6) inbred mice and MMP-12 deficient mice (all 5-6 weeks of age) were purchased from The Jackson Labs (JAX, Bar Harbor, ME). The same B6 mice served as the background strain for *MMP-12*^*-/-*^ mice. All mice were housed in an animal facility at the Johns Hopkins Asthma and Allergy Center. The room was maintained at a temperature of 21 ± 1°C (mean ± SEM) and with a 12-h light/dark cycle. Regular rodent chow and tap water were provided ad libitum. All experiments were conducted with approval from the Animal Care and Use Committee of the Johns Hopkins University Medical Institutions.

### Left pulmonary artery ligation (LPAL)

Surgery was performed as previously described [2,3]. Mice were anesthetized (2% isoflurane in air), intubated, and ventilated with the anesthetic/gas mixture. A left lateral thoracotomy was performed (third intercostal space), the left pulmonary artery was separated from the airway and ligated with silk suture. The thoracotomy was closed and mice were allowed to recover. Surgical control mice (sham) were treated the same as experimental mice in all respects except they lacked LPAL. Naïve mice were euthanized by cervical dislocation as were all other mice at specific time points for tissue harvest or evaluated 14 days after LPAL for functional angiogenesis (blood flow determination).

### Real time RT-PCR

Using standard techniques, total RNA isolation was prepared from left lungs from each group of mice (n = 3 mice per strain/time points: naïve, 3 d, 14 d) using the TRIzol reagent (Invitrogen, Carlsbad, CA) following the manufacturer’s instructions. Total RNA was isolated using the RNeasy Mini Kit (Qiagen, Valencia, CA), and 3 μg of total RNA was reverse transcribed to complementary DNA (cDNA) using random primers and MultiScribe reverse transcriptase (Applied Biosystems, Foster City, CA). Using 100 ng of cDNA as a template, quantification was performed by an ABI Prism 7000 Sequence Detector (Applied Biosystems) using the TaqMan 5' nuclease activity from the TaqMan Universal PCR Master Mix, fluorogenic probes (Applied Biosystems) and oligonucleotide primers (Invitrogen). TaqMan assays were repeated twice for each of 15 selected genes related to lung extracellular matrix in each lung sample. The selected genes were based on previous published results [16,17] and included: procollagen I (Col1a1), III (Col3a1), and VI (Col6a3), elastin (Eln), fibrillin 1 (Fbn1) and fibronectin 1 (Fn1). Protease genes included: matrix metalloproteinase 2 (MMP-2), 9 (MMP-9), 12 (MMP-12), and 14 (MMP-14) and cathepsin K (Ctsk). Anti-protease genes included: tissue inhibitor of metalloproteinase 1 (Timp1), 2 (Timp2), 3 (Timp3) and 4 (Timp4). The mRNA expression levels of all samples were normalized to the levels for the housekeeping gene glyceraldehyde-3-phosphate dehydrogenase (GAPDH) from the same sample, and relative fold changes were calculated using the 2-ΔΔCT method [16,17]. To confirm B6 and D2 strain differences in naïve lung tissue, results of each gene for D2 mice were reported as fold-changes referenced to B6 mice.

### MMP-12 activity

The time course of MMP-12 activity was measured in B6 (n = 3 mice/time point) and D2 (n = 3 mice/time points: 0 hr, 7 d, 10 d, 14 d) left lung tissue lysates using the Sensolyte 520 MMP-12 assay kit (Anaspec, San Jose, California, USA) according to the manufacturer’s instructions. Fluorescence, resulting from enzyme-mediated conversion of the fluorogenic substrates, was measured in a Novostar plate reader (BMG Labtech, Durham, NC) using black 96-well plates (Corning, Corning, NY) at excitation/emission wavelengths of 490/520 nm respectively. Substrate-only samples were included for background subtraction purposes. All measurements were performed in duplicate.

### MMP-12 lung cell localization

The cell source of MMP-12 was evaluated using immunostaining followed by flow cytometry and histochemistry. Freshly extracted left lung mouse tissue (n = 3/time point/strain) was minced and digested using previously described methods [22]. Nonspecific binding sites were blocked (Fc Block; BD Pharmingen, San Jose, CA), cells were incubated with a pan macrophage marker (APC labeled F4/80; Ebioscince, San Diego, CA), and LIVE/DEAD® Fixable Blue fluorescent reactive dye (Invitrogen, Carlsbad, CA) and fixed, permeabilized, and stained with a monoclonal antibody for MMP-12 which identifies 3 isoforms (54/45/22 kDA MW; Epitomics, Burlingame, CA ). Flow cytometry was performed using a FACSAria (Becton Dickinson, Franklin Lanes, NJ), and data analyzed with FlowJo software (Treestar Inc, Ashland, OR). In a separate series of mice (n = 2/time point/strain), lungs were inflated with Optimal Cutting Temperature Compound (OCT), frozen in OCT and sectioned. Frozen sections were blocked and stained with antibody against MMP-12 and F4/80 and 4, 6-diamidino-2-phenylindole (DAPI, Invitrogen, Carlsbad, CA). Sections were visualized and photographed using an Olympus IX51 microscope and SensiCam high performance digital camera (Cooke, Auburn Hills, MI).

### Protein evaluation

Changes in lung MIP-2α protein were evaluated in B6 mice (n = 6), D2 mice (n = 4), and *MMP-12*^*-/-*^ mice (n = 6). Twenty-four hrs after LPAL, anesthetized mice were euthanized, and the upper left lung and the upper right lung were rapidly excised and frozen. This time point was selected based on previous results demonstrating that chemokine expression was maximum and trended downward at later time points [3]. Lung samples were weighed, homogenized, and aliquoted. MIP-2α was determined by ELISA (Duoset Mouse MIP-2, ELISA kit; R&D Systems, Minneapolis, MN) and normalized to total sample protein (BCA protein assay kit; Pierce, Rockford, IL).

Angiostatin was measured in additional mice (6 mice/B6 and D2 mice, 3 MMP-12 mice) 14 days after LPAL. This time point was selected based on late changes in MMP-12 activity and the expectation that an angiostatic factor would be most abundant late after LPAL. Lungs were obtained as described above, angiostatin determined by ELISA (Angiostatin ELISA kit; USCN Life Science, Wuhan, China) and normalized to total sample protein (BCA protein assay kit; Pierce, Rockford, IL).

### Angiogenesis assessment

After mice were anesthetized and intubated, the extent of neovascularization was determined by measuring systemic blood flow to the left lung 14 days after LPAL using fluorescent microspheres (n = 4-5/group; Invitrogen, Eugene, OR) [23]. The carotid artery was cannulated and 360,000 microspheres (10 μm) were infused with a pump. Mice were sacrificed by exsanguination and the left lung was excised. Microspheres lodged in the left lung were quantified after tissue digestion and fluorescent dye extraction, and the number calculated from a standard curve. Data are presented as the number of microspheres delivered to the left lung relative to total infused (functional angiogenesis, %).

### Statistical analysis

The results reported in the figures and tables are means (± SEM). For RT-PCR values, multiple Student t-tests were performed to establish significance between group means, and were considered statistically significant at p < 0.01. For all other analyses, p < 0.05 was the accepted significance level. Angiogenesis was evaluated by ANOVA followed by Newman-Keuls Comparison of means. A 2-way ANOVA was used to evaluate time dependent changes in MMP-12 activity, MMP-12+ F4/80+ cells and MIP-2α levels.

## Results

### Strain variation in gene expression profiles

To confirm baseline differences in select lung structural, protease and anti-protease genes between D2 and B6 mice, we measured gene expression profiles in naïve lung tissue (Table 1). In this analysis we applied the criteria of requiring at least a two-fold increase at a significance level of p < 0.01. As compared to levels in B6 mice, the gene expression in D2 mice was significantly lower for the *Eln* gene. The expression levels of *MMP-2* and *MMP-12* genes were significantly higher in D2 mice by more than respectively, 20- and 3-fold on average relative to B6 mice. There were no detectable differences in anti-protease gene expression profiles between strains. These results confirm some previous observations [16,17] suggesting inherent differences in the expression of several genes between D2 and B6 mouse strains that might influence lung structure. To determine whether gene expression profiles changed after the onset of ischemia, left lungs from D2 and B6 mice were studied 3 days after LPAL, a time of active neovascularization [24] and again 14 days after LPAL when a neovasculature is fully established [25]. Table 1 shows matrix gene expression relative to naïve B6 lungs, 3 days and 14 days after LPAL. Both strains showed a significant increase in *Eln* 14 days after LPAL. However, with regard to protease genes, *MMP-12* and *MMP-14* were significantly up-regulated in LPAL B6 mice and not in D2 mice 3 days after LPAL. The anti-protease genes were more substantially upregulated in D2 mice after ischemia compared with B6 mice. Mice undergoing sham surgery showed no significant change from naïve lungs (data not shown).

**Table 1:** Changes in matrix protein gene expression in B6 vs D2 mice

	**B6**		**D2**		
	**3d LPAL**	**14d LPAL**	**Naïve**	**3d LPAL**	**14d LPAL**
**Structural genes**					
Col1a1	1.3 ± 0.1	1.4 ± 0.2	1.1 ± 0.1	2.1 ± 0.6	1.6 ± 0.2
Col3a1	1.4 ± 0.0	1.2 ± 0.2	1.6 ± 0.0	1.7 ± 0.1	1.7 ± 0.2
Col6a3	1.3 ± 0.1	1.7 ± 0.2	1.1 ± 0.2	1.7 ± 0.4	2.2 ± 0.5
Eln	1.8 ± 0.2	3.0 ± 0.5*	0.5 ± 0.1*	2.4 ± 0.8	**3.5** ± 0.5*
Fbn1	1.1 ± 0.2	1.1 ± 0.1	0.9 ± 0.1	2.2 ± 1.0	1.4 ± 0.1
Fn1	1.3 ± 0.1	1.3 ± 0.2	1.3 ± 0.3	1.5 ± 0.2	1.3± 0.1
**Protease genes**					
MMP-2	1.0 ± 0.1	1.9 ± 0.2*	20.1 ± 1.0*	0.9 ± 0.2	1.5 ± 0.1
MMP-9	1.3 ± 0.2	0.5 ± 0.2	0.9 ± 0.1	1.3 ± 0.3	0.4 ± 0.0*
MMP-12	3.6 ± 0.7*	1.7 ± 0.1	3.2 ± 0.5*	2.0 ± 0.8	2.9 ± 1.2
MMP-14	2.0 ± 0.1*	1.5 ± 0.2	1.0 ± 0.1	2.7 ± 0.8	2.0 ± 0.3
Ctsk	1.3 ± 0.1	1.8 ± 0.3	1.6 ± 0.1	1.1 ± 0.2	1.7 ± 0.2
**Anti-protease genes**					
Timp1	8.8 ± 0.6*	1.5 ± 0.2	0.8 ± 0.0	**19.6** ± 2.9*	**3.8** ± 0.1*
Timp2	0.7 ± 0.1	0.9 ± 0.0	1.5 ± 0.1	0.8 ± 0.2	1.5 ± 0.1
Timp3	0.7 ± 0.1	1.0 ± 0.0	1.7 ± 0.2	1.1 ± 0.3	**2.4** ± 0.2*
Timp4	0.5 ± 0.1	0.6 ± 0.1	0.6 ± 0.1	1.2 ± 0.1	**2.5** ± 0.4*

Expression of matrix-associated genes (* indicates p < 0.01). All comparisons were made versus B6 naïve left lungs.

### MMP-12 lung cell localization

**Figure 1:**
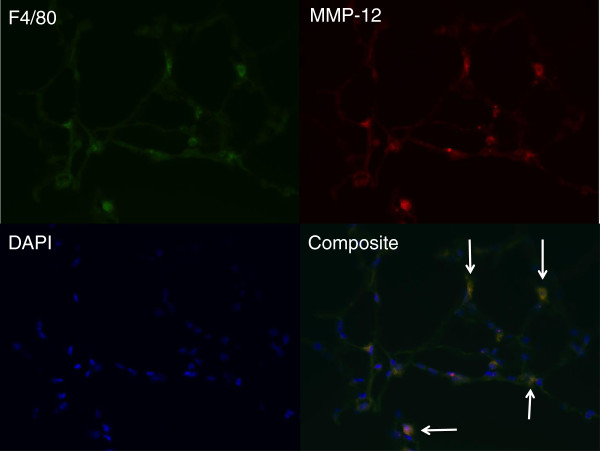
**Histologic section of left lung from C57 mouse 14 d after LPAL (400 x magnification).** MMP-12+ cells (red) are co-localized with F4/80+ macrophages (green). DAPI (blue) was used to show all cell nuclei. Composite shows co-localization of staining with arrows indicating several of the MMP-12+ F4/80+ cells.

Figure 1 shows a representative frozen section of a left lung 14 d after LPAL from a B6 mouse (400x magnification). Prominent staining of F4/80+ macrophages shows co-localization with MMP-12+ cells within the lung parenchyma. The antibody to MMP-12 recognized both active and inactive isoforms of MMP-12. Extensive review of histologic sections did not reveal any consistent MMP-12+ F4/80- cells. Quantification of strain and time-dependent association of MMP-12+ F4/80+ cells in the lung after LPAL was analyzed in additional mice. Flow cytometric analysis of dispersed mouse lungs (n = 3 mice/strain/time point) demonstrated that MMP-12 was predominantly associated with F4/80+ macrophages in B6 (80 ± 6%) and D2 mice (78 ± 4%) at the onset of ischemia (0 hr). These values did not alter when measured 14 d after LPAL in B6 (74 ± 7%) or D2 mice (74 ± 25%).

### MMP-12 activity

**Figure 2:**
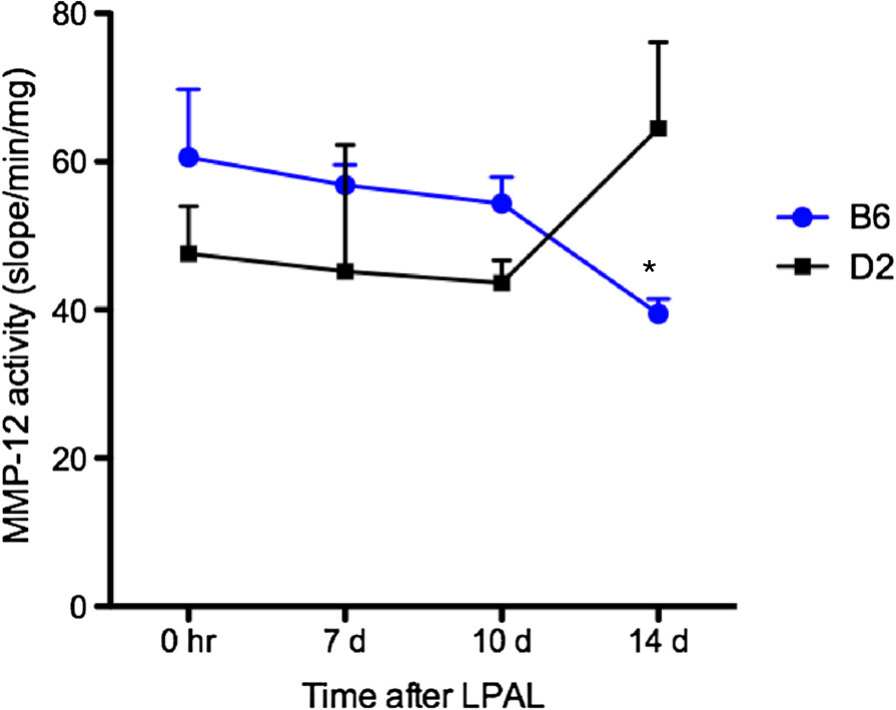
**MMP-12 activity (slope/min/mg) over the 14 d time course after ischemia in B6 and D2 mice (n = 3 mice/time point).** MMP-12 activity in B6 mice decreases with time, while it increases with time in D2 mice. This time-dependent change in MMP-12 activity is significantly different between strains; p < 0.05.

Because of the baseline differences observed in *MMP-12* expression and early increase in MMP-12 expression in ischemic B6 mice, the time course of MMP-12 protease activity was measured in the two strains. Figure 2 shows the slow decline of MMP-12 activity in B6 mice and the slow increase in MMP-12 activity in D2 mice over the course of 14 days after LPAL. This time-dependent change in MMP-12 activity was statistically different between strains (F-stat = 3.43, p = 0.047).

### Strain variation in angiogenesis

**Figure 3:**
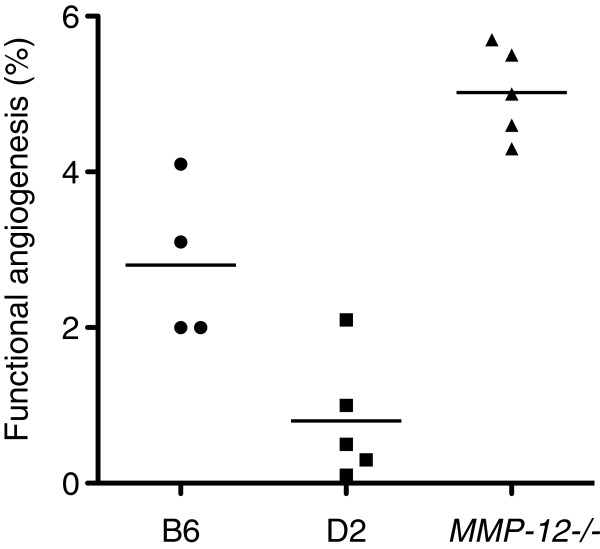
**Functional angiogenesis (% cardiac output) in three mouse strains.** Changes in lung neovascularization were assessed by measuring left lung perfusion 14 d after LPAL. Each point represents one mouse. Each group was significantly different from the other with angiogenesis greatest in *MMP-12*^-/-^, followed by B6, and the lowest level was observed in D2 mice (p < 0.01 for all comparisons).

Changes in systemic neovascularization of the left lung 14 days after LPAL are shown in Figure 3. Functional angiogenesis evaluated by systemic perfusion of the left lung is shown for individual B6, D2, and *MMP-12*^*-/-*^ mice (p < 0.001). This functional assessment of angiogenesis in B6 mice showed an average systemic perfusion to the left lung of 2.8 ± 0.5% of total cardiac output, which was equivalent to previously reported values for this time point after LPAL [1,2]. This index of functional angiogenesis was significantly higher in B6 mice compared to D2 mice which averaged 0.8 ± 0.4% of cardiac output (p < 0.01). Furthermore, angiogenic perfusion to the left lung of *MMP-12*^*-/-*^ mice 14 days after LPAL was significantly greater than both B6 and D2 mice averaging 5.0 ± 0.3% (p < 0.01). Thus, the collective strain variation in functional angiogenesis among the three groups shows that neovascularization is the lowest in D2 mice and the highest in *MMP-12*^*-/-*^ mice.

### Protein

**Figure 4:**
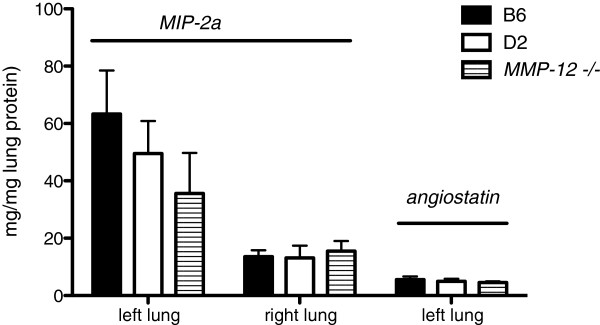
**MIP-2**α **protein and angiostatin in lung homogenates after LPAL in the three mouse strains normalized to total lung protein.** A significant increase in MIP-2α protein was observed 24 hrs after LPAL in left lungs of B6 and D2 mice relative to paired right lungs (p < 0.05). No strain-dependent differences in left lungs were observed (n = 4-6 mice /group). No strain-dependent differences were observed in the level of left lung angiostatin 14 d after LPAL (6 mice B6 and D2 mice, 3 MMP-12 mice).

Left lung homogeneate was assessed 24 hrs after LPAL to determine if strain-dependent differences existed in the level of the proangiogenic chemokine, MIP-2α. Left lung values are compared to paired right lung controls in B6, D2, and *MMP-12*^*-/-*^ mice and are shown in Figure 4. As previously reported, there was a significant difference in left lung MIP-2α protein versus right lung in B6 mice [26]. This difference was also evident in D2 mice. However, there were no significant differences among left lungs of the three strains or among right lungs at this time point after LPAL.

Lung homogenates were assayed 14 days after LPAL to determine if strain-dependent differences existed in the level of the MMP-12-dependent anti-angiogenic protein angiostatin. No significant differences between left and right lung angiostatin values were seen, nor were there differences in the left lungs across strains (Figure 4). Angiostatin level for left lungs of B6 mice averaged 5.6 ± 1.1 μg/mg lung protein, for D2 mice: 5.0 ± 0.9 μg/mg lung protein, and for *MMP-12*^*-/-*^ mice: 4.6 ± 0.4 μg/mg lung protein.

## Discussion

The role of matrix proteins during angiogenesis has been studied yet results demonstrate that key regulators are highly organ-specific [27]. Angiogenic growth factors are unique to local environmental conditions. In the ischemic, ventilated lung, hypoxia-inducible factors appear not to play a major role in systemic vascularization whereas inflammatory cytokines have been shown to be critical to the process of new vessel growth [3]. Several metalloproteinases have been implicated in the metabolism of growth factors [7-9]. However, the importance of matrix proteins in the process of systemic neovascularization of the lung has not been reported. In the present study we relied on previously reported, strain-dependent differences in matrix proteins to probe the importance of select proteases to the process of systemic neovascularization. Our results identify MMP-12 as an angiostatic factor in the ischemic lung that appears to modulate the extent of systemic neovascularization in this non-hypoxic environment. However, the specific mechanisms by which the activity of MMP-12 limits neovascularization remain unclear.

Ischemic injury in the lung has been shown to result in permeability changes [25], inflammation [26], and subsequent systemic neovascularization of the lung [25,26]. Systemic angiogenesis during pulmonary vascular ischemia includes the growth of bronchial and intercostal arteries and has been documented in dogs [28,29], sheep [30], rats [31], mice [1], and in patients with chronic pulmonary thromboembolism [32]. Yet the complex process of tissue repair including the tunneling of proliferating vessels is not known. New endothelial networks are established in response to chemotactic stimuli. Within the lung, this repair likely includes endothelial proliferation and reorganization of existing cells and structures. To begin to dissect this complex process, we first surveyed candidate genes known to be involved in other forms of lung injury and we studied them in two previously reported, divergent mouse strains [16,17]. We also measured angiogenesis in these two strains and found that D2 mice showed a significantly reduced angiogenic phenotype compared with B6 mice (Figure 3). Systemic perfusion of the left lung 14 days after the onset of total left lung ischemia in D2 mice was on average, 30% of that observed in B6 mice. Based on this finding and previously published data demonstrating the time course of neovascularization [1], we surveyed matrix gene expression profiles 3 days after the onset of ischemia when active changes were expected and compared them to expression levels 14 days after LPAL when a new network was established. Of the 15 genes surveyed, MMP-12 was confirmed to be significantly greater in D2 mice than in B6 mice at baseline in naïve mice. Furthermore, MMP-12 expression increased in B6 mice by 3 days. Although a complete time course was not obtained, we showed that MMP-12 was changing, it was expressed differently but in both strains, and Timp1, the anti-protease regulator of MMP-12 increased in both strains. Thus, we hypothesized that MMP-12 exerted an important influence on systemic angiogenesis in the lung during ischemia. While transcription profiles appeared to be altered by ischemia, it was not clear that they would be predictive of protein activity. Additionally, others had shown MMP-12 altered the metabolism of growth factors [7] as well as angiostatic factors [10]. Subsequent experiments focused specifically on the effects of MMP-12 in this model.

In an effort to evaluate the functional relevance of the changes in gene expression, we measured the time course of MMP-12 activity after ischemia in the lung. No differences were seen in MMP-12 activity between the D2 and B6 mice at the 0 hr time point (immediately after LPAL). However, incremental decreases in MMP-12 activity were observed over the course of 14 days only in B6 mice. D2 mice showed a small increase in MMP-12 activity by 14 days after LPAL. Statistical analysis showed a significant interaction between the effects of time and strain from immediately after the onset of ischemia to the time when a functional systemic bed was established. Over the course of 14 days, MMP-12 activity differed between the 2 mouse strains. The activity profile did not directly parallel gene expression pattern yet was consistent with the hypothesis that MMP-12 activity might contribute to the decrease in angiogenesis observed in D2 compared to B6 mice in this ischemic model.

In an effort to localize the predominant source of MMP-12 in the lung, we used immunohistochemistry and flow cytometry. Histologic sections demonstrated co-localization of MMP-12 with F4/80+ macrophages. No significant differences were seen in the percent of live cells in the lung expressing MMP-12 and F4/80 between strains or over time. However, the antibody used for MMP-12 recognized both active and inactive isoforms of MMP-12 and likely accounts for this observation. Furthermore, it highlights the overall importance of measuring the activity of MMP-12 throughout the time course of ischemia.

Studying angiogenesis in mice genetically deficient in MMP-12 provided a phenotype for comparison to D2 and B6, 14 days after LPAL. Figure 3 shows the difference in functional angiogenesis in *MMP-12*^*-/-*^ mice compared to the two other strains. *MMP-12*^*-/-*^ mice exhibited an 80% increase in perfusion of the left lung relative to the B6 background strain. This observation, that MMP-12 deficiency allowed for greater perfusion and neovascularization, is consistent with the MMP-12 activity data in the two strains. The greatest MMP-12 activity is associated with the lowest angiogenic outcome. These findings establish that MMP-12 is overall angiostatic in the ischemic lung.

Two series of experiments were performed in an attempt to determine the mechanism by which MMP-12 activity might impact angiogenesis in the ischemic mouse lung. The first series was based on the findings of Dean and colleagues that demonstrated that MMP-12 was critical for the metabolism and inactivation of both MIP-2α and KC [7]. Previous work from our laboratory has shown these chemokines to be prominent, pro-angiogenic growth factors that increased by 24 hrs after the onset of lung ischemia [3]. Therefore, we measured MIP-2α at this single time point in the three mouse strains. Our results confirmed significant differences between left and right lungs but no differences between strains. This lack of difference between D2 and B6 mice was consistent with the observation of similar MMP-12 activity levels initially at the onset of ischemia. Additionally, *MMP-12*^*-/-*^ mice showed no significant difference in MIP-2α levels suggesting that this proteolytic function is not operative in the lung or that MIP-2α levels exceeded the capacity of this metabolic pathway at this time point.

Since there was a slow decline in MMP-12 activity in the B6 mice and an increased level in the D2 mice over the course of 14 days of ischemia, we speculated in a second series of experiments that increased MMP-12 activity contributed directly to an angiostatic protein that slowed angiogenesis in D2 mice. Cornelius and coworkers showed that MMP-12 is essential for the formation of angiostatin, an important anti-angiogenic by-product of plasminogen metabolism, which inhibits endothelial cell proliferation [10]. Other clinical studies have arrived at the same conclusion regarding the inhibitory role MMP-12 plays in hindering angiogenesis [10,33,34]. Thus we measured angiostatin levels in the lungs of all three strains 14 days after LPAL. We reasoned that at this time point we should see differences in levels if angiostatin had a slowing effect. Results showed similar levels in left and right lungs of all mice. Thus, we concluded that either the methods to measure angiostatin (ELISA) were inadequate, the time point selected was not optimal for angiostatin detection, or angiostatin is not important in this model.

We focused our studies on the role of MMP-12 during ischemia-induced angiogenesis since this metalloproteinase has been reported to be involved with the metabolism of growth/angiostatic factors. However, there were large significant differences in Eln (decreased), MMP-2 (increased) in D2 naïve lungs relative to B6 naïve lungs, and increases in Timp1 during ischemia in both strains. Each has been associated with changes in vascularization and might play a regulatory role during ischemia. Regulated elastin production and deposition have been shown to be critical for normal neonatal vasculogenesis of the pulmonary vasculature [35]. Supportive of the finding that lung structural components impact angiogenesis during ischemia, our laboratory showed hyaluronan fragments significantly increase systemic angiogenesis in the adult [23]. Additionally, the role of Timp1 has been shown to be anti-angiogenic in a variety of models largely through the inhibition of pro-angiogenic MMP [7,36-38]. Thus, these and other matrix-associated gene products require further evaluation in the ischemic lung.

## Conclusion

In summary, we used gene expression profiling of a panel of matrix-specific genes, angiogenesis quantification, and divergent mouse strains to identify gene products that may play a role in directing the course of systemic neovascularization of the lung after ischemic injury. Our results show that MMP-12 is angiostatic in this ischemic, non-hypoxic lung injury model. Strategies to activate this metalloproteinase may provide therapeutic opportunities to prevent excessive neovascularization in inflammatory lung disease.

## Competing interests

The authors declare that they have no competing interests.

## Authors’ contributions

CT acquired and analyzed gene expression data, AM performed all immunohistochemistry and bioassays, and EW coordinated experiments, interpreted all results and prepared the manuscript. All authors read and approved the final manuscript.
